# Supplementary material for: Association of the COVID‐19 lockdown with smoking, drinking and attempts to quit in England: an analysis of 2019–20 data
Source: Addiction. 2020 Nov 26;116(5):1233–44. doi: 10.1111/add.15295 (PMC8436745; doi:10.1111/add.15295)
Supplement: Supplementary file 1 — File S1 Smoking and drinking outcomes in relation to covariates [file ADD-116-1233-s002.docx]

**Supplementary File 1: smoking and drinking outcomes in relation to covariates**

| **Supplementary Table 1.** Smoking and quitting in England before (April 2019–February 2020) versus after (April 2020) the Covid-19 pandemic in relation to sociodemographic variables | | | | | | | | | | | | | | | | | | | | | | | | | | | | | | | | | | | | | | | | | | | |
| --- | --- | --- | --- | --- | --- | --- | --- | --- | --- | --- | --- | --- | --- | --- | --- | --- | --- | --- | --- | --- | --- | --- | --- | --- | --- | --- | --- | --- | --- | --- | --- | --- | --- | --- | --- | --- | --- | --- | --- | --- | --- | --- | --- |
|  | | | **Smoking prevalence^1^**  **(%)** | | | |  | | **Cessation**^2^  **(%)** | | |  | **Quit attempts**^2^  **(%)** | | | | | |  | | | | | **Quit success^3^**  **(%)** | | | | | | |  | **Use of evidence-**  **based support^3a^**  **(%)** | | | |  | | | **Use of remote support^3b^**  **(%)** | | |  |  |
|  | | | **Before** | | **After** | |  | | **Before** | | **After** |  | **Before** | **After** | | | |  | | | | | **Before** | | | | | | **After** | |  | **Before** | | | **After** | |  | | | **Before** | **After** | |  |
| Age in years | | |  | |  | |  | |  | |  |  |  |  | | | |  | | | | |  | | | | | |  | |  |  | | |  | |  | | |  |  | |  |
|  | 16-24 | | 19.3 | | 23.1 | |  | | 5.4 | | 15.1 |  | 27.5 | 64.2 | | | |  | | | | | 18.0 | | | | | | 23.5 | |  | 35.7 | | | 41.2 | |  | | | 4.3 | 0.0 | |  |
|  | 25-34 | | 22.9 | | 26.7 | |  | | 3.9 | | 14.4 |  | 31.2 | 47.1 | | | |  | | | | | 12.3 | | | | | | 31.7 | |  | 44.1 | | | 57.1 | |  | | | 0.8 | 19.0 | |  |
|  | 35-44 | | 16.6 | | 23.2 | |  | | 5.4 | | 2.9 |  | 31.3 | 31.3 | | | |  | | | | | 16.8 | | | | | | 9.5 | |  | 55.6 | | | 45.5 | |  | | | 5.0 | 22.7 | |  |
|  | 45-54 | | 16.4 | | 16.5 | |  | | 3.4 | | 1.9 |  | 30.0 | 27.5 | | | |  | | | | | 11.9 | | | | | | 6.7 | |  | 60.2 | | | 50.0 | |  | | | 3.1 | 7.1 | |  |
|  | 55-64 | | 14.7 | | 12.6 | |  | | 3.1 | | 5.9 |  | 27.1 | 30.3 | | | |  | | | | | 11.7 | | | | | | 20.0 | |  | 63.1 | | | 60.0 | |  | | | 0.9 | 0.0 | |  |
|  | ≥65 | | 8.3 | | 6.2 | |  | | 3.0 | | 9.7 |  | 24.2 | 20.7 | | | |  | | | | | 11.6 | | | | | | 16.7 | |  | 58.1 | | | 50.0 | |  | | | 3.5 | 0.0 | |  |
| Sex | | |  | |  | |  | |  | |  |  |  |  | | | |  | | | | |  | | | | | |  | |  |  | | |  | |  | | |  |  | |  |
|  | Male | | 17.3 | | 19.1 | |  | | 4.3 | | 7.3 |  | 28.9 | 39.1 | | | |  | | | | | 15.2 | | | | | | 17.9 | |  | 51.3 | | | 50.0 | |  | | | 2.5 | 11.8 | |  |
|  | Female | | 14.5 | | 14.9 | |  | | 3.9 | | 10.6 |  | 29.2 | 40.4 | | | |  | | | | | 12.4 | | | | | | 25.4 | |  | 51.7 | | | 50.8 | |  | | | 3.1 | 10.0 | |  |
| Social grade | | |  | |  | |  | |  | |  |  |  |  | | | |  | | | | |  | | | | | |  | |  |  | | |  | |  | | |  |  | |  |
|  | ABC1 | | 10.4 | | 11.4 | |  | | 5.8 | | 8.4 |  | 31.8 | 39.0 | | | |  | | | | | 17.6 | | | | | | 21.7 | |  | 44.9 | | | 37.0 | |  | | | 2.4 | 10.9 | |  |
|  | C2DE | | 22.6 | | 23.9 | |  | | 3.0 | | 9.3 |  | 27.4 | 39.4 | | | |  | | | | | 11.3 | | | | | | 22.8 | |  | 56.1 | | | 57.7 | |  | | | 3.1 | 10.3 | |  |
| Region in England | | |  | |  | |  | |  | |  |  |  |  | | | |  | | | | |  | | | | | |  | |  |  | | |  | |  | | |  |  | |  |
|  | London | | 14.7 | | 17.1 | |  | | 4.7 | | 0.0 |  | 34.1 | 34.0 | | | |  | | | | | 13.7 | | | | | | 0.0 | |  | 41.4 | | | 29.4 | |  | | | 2.6 | 17.6 | |  |
|  | South | | 14.6 | | 16.4 | |  | | 4.8 | | 7.1 |  | 31.8 | 36.7 | | | |  | | | | | 14.0 | | | | | | 20.7 | |  | 47.1 | | | 44.8 | |  | | | 2.1 | 10.3 | |  |
|  | Central | | 16.4 | | 16.6 | |  | | 2.7 | | 10.1 |  | 25.4 | 40.8 | | | |  | | | | | 10.6 | | | | | | 22.5 | |  | 52.2 | | | 57.5 | |  | | | 2.4 | 2.5 | |  |
|  | North | | 17.2 | | 17.9 | |  | | 4.7 | | 13.5 |  | 28.1 | 43.6 | | | |  | | | | | 16.9 | | | | | | 31.0 | |  | 60.8 | | | 56.1 | |  | | | 3.9 | 16.7 | |  |
| Heaviness of smoking index score | | |  | |  | |  | |  | |  |  |  |  | | | |  | | | | |  | | | | | |  | |  |  | | |  | |  | | |  |  | |  |
|  | <4 | | - | | - | |  | | 3.6 | | 9.1 |  | 28.9 | 39.5 | | | |  | | | | | 12.2 | | | | | | 21.4 | |  | 50.6 | | | 47.6 | |  | | | 2.5 | 8.7 | |  |
|  | ≥4 | | - | | - | |  | | 4.4 | | 10.0 |  | 28.1 | 35.5 | | | |  | | | | | 15.8 | | | | | | 30.0 | |  | 62.1 | | | 81.8 | |  | | | 3.2 | 0.0 | |  |
| Note: All data are weighted to match the adult population in England on age, social grade, region, tenure, ethnicity, and working status within sex.  ^1^ Among all adults. ^2^ Among past-year smokers. ^3^ Among past-year smokers who made a quit attempt.  ^a^ Prescription medication, face-to-face behavioural support, nicotine replacement therapy obtained over the counter, e-cigarettes.  ^b^ Telephone support, websites, or apps. | | | | | | | | | | | | | | | | | | | | | | | | | | | | | | | | | | | | | | | | | | | |
|  | | | | | | | | | | | | | | | | | | | | | | | | | | | | | | | | | | | | | |  |  |  |  |  |  |
| **Supplementary Table 2.** High-risk drinking and alcohol reduction attempts in England before (April 2019–February 2020) versus after (April 2020) the Covid-19 pandemic in relation to sociodemographic variables | | | | | | | | | | | | | | | | | | | | | | | | | | | | | | | | |  |  |  |  |  |  |  |  |  |  |  |
|  | | **High-risk drinking prevalence^1^**  **(%)** | | | |  | | **Alcohol reduction attempts**^2^  **(%)** | | | |  | **Use of evidence-**  **based support^3a^**  **(%)** | | | | | | | |  | | | | | **Use of remote support^3b^**  **(%)** | | | | | | |  |  |  |  |  |  |  |  |  |  |  |
|  | | **Before** | | **After** | |  | | **Before** | | **After** | |  | **Before** | | **After** | | | | | | |  | | | | | **Before** | | | **After** | | |  |  |  |  |  |  |  |  |  |  |  |
| Age in years | |  | |  | |  | |  | |  | |  |  | |  |  |  | | |  | | | | |  | | |  | | | | |  |  |  |  |  |  |  |  |  |  |  |
|  | 16-24 | 32.0 | | 42.5 | |  | | 9.6 | | 22.7 | |  | 2.6 | | 5.9 | | | | | | |  | | | | | 6.6 | | | 0.0 | | |  |  |  |  |  |  |  |  |  |  |  |
|  | 25-34 | 24.4 | | 38.8 | |  | | 15.4 | | 33.3 | |  | 4.3 | | 0.0 | | | | | | |  | | | | | 5.2 | | | 5.7 | | |  |  |  |  |  |  |  |  |  |  |  |
|  | 35-44 | 26.8 | | 43.9 | |  | | 16.2 | | 24.6 | |  | 2.4 | | 3.6 | | | | | | |  | | | | | 13.7 | | | 24.1 | | |  |  |  |  |  |  |  |  |  |  |  |
|  | 45-54 | 28.7 | | 48.2 | |  | | 19.4 | | 29.7 | |  | 5.8 | | 0.0 | | | | | | |  | | | | | 7.0 | | | 5.4 | | |  |  |  |  |  |  |  |  |  |  |  |
|  | 55-64 | 27.6 | | 35.7 | |  | | 19.1 | | 33.7 | |  | 3.6 | | 0.0 | | | | | | |  | | | | | 0.0 | | | 3.4 | | |  |  |  |  |  |  |  |  |  |  |  |
|  | ≥65 | 15.8 | | 26.8 | |  | | 11.5 | | 26.3 | |  | 4.0 | | 0.0 | | | | | | |  | | | | | 2.7 | | | 0.0 | | |  |  |  |  |  |  |  |  |  |  |  |
| Sex | |  | |  | |  | |  | |  | |  |  | |  | | | | | | |  | | | | |  | | |  | | |  |  |  |  |  |  |  |  |  |  |  |
|  | Male | 33.5 | | 46.0 | |  | | 14.5 | | 25.3 | |  | 2.1 | | 0.0 | | | | | | |  | | | | | 6.4 | | | 6.6 | | |  |  |  |  |  |  |  |  |  |  |  |
|  | Female | 16.9 | | 30.9 | |  | | 16.8 | | 33.2 | |  | 7.1 | | 2.4 | | | | | | |  | | | | | 5.3 | | | 8.4 | | |  |  |  |  |  |  |  |  |  |  |  |
| Social grade | |  | |  | |  | |  | |  | |  |  | |  | | | | | | |  | | | | |  | | |  | | |  |  |  |  |  |  |  |  |  |  |  |
|  | ABC1 | 30.5 | | 42.2 | |  | | 16.5 | | 30.2 | |  | 2.0 | | 0.9 | | | | | | |  | | | | | 4.5 | | | 9.1 | | |  |  |  |  |  |  |  |  |  |  |  |
|  | C2DE | 18.4 | | 33.8 | |  | | 12.9 | | 25.4 | |  | 9.3 | | 1.7 | | | | | | |  | | | | | 9.8 | | | 3.4 | | |  |  |  |  |  |  |  |  |  |  |  |
| Region in England | |  | |  | |  | |  | |  | |  |  | |  | | | | | | |  | | | | |  | | |  | | |  |  |  |  |  |  |  |  |  |  |  |
|  | London | 22.8 | | 37.3 | |  | | 26.8 | | 31.9 | |  | 3.5 | | 0.0 | | | | | | |  | | | | | 4.7 | | | 17.2 | | |  |  |  |  |  |  |  |  |  |  |  |
|  | South | 24.6 | | 36.7 | |  | | 14.7 | | 36.7 | |  | 5.2 | | 0.0 | | | | | | |  | | | | | 6.9 | | | 1.7 | | |  |  |  |  |  |  |  |  |  |  |  |
|  | Central | 22.4 | | 34.0 | |  | | 13.0 | | 23.4 | |  | 4.3 | | 0.0 | | | | | | |  | | | | | 6.7 | | | 5.3 | | |  |  |  |  |  |  |  |  |  |  |  |
|  | North | 29.6 | | 44.9 | |  | | 12.9 | | 24.5 | |  | 3.6 | | 4.2 | | | | | | |  | | | | | 6.2 | | | 10.2 | | |  |  |  |  |  |  |  |  |  |  |  |
| AUDIT score | |  | |  | |  | |  | |  | |  |  | |  | | | | | | |  | | | | |  | | |  | | |  |  |  |  |  |  |  |  |  |  |  |
|  | ≤7 | - | | - | |  | | 9.6 | | 20.6 | |  | 1.7 | | 0.0 | | | | | | |  | | | | | 2.6 | | | 0.0 | | |  |  |  |  |  |  |  |  |  |  |  |
|  | 8-19 | - | | - | |  | | 20.5 | | 35.3 | |  | 2.6 | | 1.0 | | | | | | |  | | | | | 5.9 | | | 10.3 | | |  |  |  |  |  |  |  |  |  |  |  |
|  | ≥20 | - | | - | |  | | 53.6 | | 66.7 | |  | 28.9 | | 9.1 | | | | | | |  | | | | | 26.7 | | | 18.2 | | |  |  |  |  |  |  |  |  |  |  |  |
| Note: All data are weighted to match the adult population in England on age, social grade, region, tenure, ethnicity, and working status within sex.  ^1^ Among all adults. ^2^ Among high-risk drinkers. ^3^ Among high-risk drinkers who made a reduction attempt.  ^a^ Prescription medication or face-to-face behavioural support.  ^b^ Telephone support, websites, or apps. | | | | | | | | | | | | | | | | | | | | | | | | | | | | | | | | |  |  |  |  |  |  |  |  |  |  |  |
