# Supplementary material for: Association of the COVID‐19 lockdown with smoking, drinking and attempts to quit in England: an analysis of 2019–20 data
Source: Addiction. 2020 Nov 26;116(5):1233–44. doi: 10.1111/add.15295 (PMC8436745; doi:10.1111/add.15295)
Supplement: Supplementary file 3 — File S3 Sensitivity analysis comparing changes in smoking and drinking outcomes between May‐Feb and April in the pandemic period (2019/20) with the previous year (2018/19) [file ADD-116-1233-s003.docx]

**Supplementary File 3: sensitivity analysis comparing changes in smoking and drinking outcomes between May-Feb and April in the pandemic period (2019/20) with the previous year (2018/19)**

| **Table 1.** Month (May-Feb vs. April) x year (2018/19 vs. 2019/20) interactions for smoking outcomes | | | | | | |
| --- | --- | --- | --- | --- | --- | --- |
|  |  | **2018/19** | **2019/20** |  | **Month x year interaction** | |
|  |  | **% [95% CI]** | **% [95% CI]** |  | **OR_adj_ [95% CI]** | ***p*** |
| **Smoking prevalence^1^** | |  |  |  |  |  |
|  | May-February | 17.3 [16.7-17.9] | 15.8 [15.3-16.4] |  | - | - |
|  | April | 16.3 [14.6-18.2] | 17.0 [15.2-18.9] |  | 1.16 [0.96-1.41] | 0.119 |
| **Cessation^2^** | |  |  |  |  |  |
|  | May-February | 4.7 [4.0-5.5] | 4.2 [3.5-5.0] |  | - | - |
|  | April | 2.8 [1.2-5.5] | 8.8 [6.0-12.4] |  | 5.40 [2.09-13.99] | 0.001 |
| **Quit attempts^2^** | |  |  |  |  |  |
|  | May-February | 29.0 [27.4-30.6] | 28.9 [27.2-30.6] |  | - | - |
|  | April | 31.0 [25.7-36.9] | 39.6 [34.2-45.1] |  | 1.38 [0.96-1.98] | 0.078 |
| **Quit success^3^** | |  |  |  |  |  |
|  | May-February | 15.1 [12.8-17.6] | 14.3 [12.0-17.0] |  | - | - |
|  | April | 9.3 [4.1-17.5] | 21.3 [14.5-29.4] |  | 4.07 [1.49-11.14] | 0.006 |
| **Use of evidence-based support^3a^** | |  |  |  |  |  |
|  | May-February | 54.2 [50.9-57.5] | 51.1 [47.6-54.6] |  | - | - |
|  | April | 54.7 [43.6-65.4] | 50.0 [41.0-59.0] |  | 1.42 [0.75-2.70] | 0.284 |
| **Use of remote support^3b^** | |  |  |  |  |  |
|  | May-February | 1.7 [1.0-2.8] | 2.6 [1.6-4.0] |  | - | - |
|  | April | 3.5 [0.7-9.9] | 10.9 [6.1-17.7] |  | 1.07 [0.24-4.82] | 0.929 |
| Note: All data are weighted to match the adult population in England on age, social grade, region, tenure, ethnicity, and working status within sex.  CI, confidence interval. OR_adj_, odds ratio adjusted for age, sex, social grade, region (and, for analyses of cessation, quit success, and use of support, heaviness of smoking index), trend within year (i.e. May=1 through April=12) and trend across years (i.e. May 2018=1 through April 2020=24).  ^1^ Among all adults (2018/19: May-Feb *n*=17,209, April *n*=1,664; 2019/20: May-Feb *n*=17,216, April *n*=1,670).  ^2^ Among past-year smokers (2018/19: May-Feb *n*=3,146, April *n*=284; 2019/20: May-Feb *n*=2,881, April *n*=329).  ^3^ Among past-year smokers who made a quit attempt (2018/19: May-Feb *n*=889, April *n*=86; 2019/20: May-Feb *n*=809, April *n*=127).  ^a^ Prescription medication, face-to-face behavioural support, nicotine replacement therapy obtained over the counter, e-cigarettes.  ^b^ Telephone support, websites, or apps. | | | | | | |

| **Table 2.** Month (May-Feb vs. April) x year (2018/19 vs. 2019/20) interactions for drinking outcomes | | | | | | |
| --- | --- | --- | --- | --- | --- | --- |
|  |  | **2018/19** | **2019/20** |  | **Month x year interaction** | |
|  |  | **% [95% CI]** | **% [95% CI]** |  | **OR_adj_ [95% CI]** | ***p*** |
| **High-risk drinking prevalence^1^** | |  |  |  |  |  |
|  | May-February | 26.2 [25.5-26.9] | 25.2 [24.6-25.9] |  | - | - |
|  | April | 23.4 [21.4-25.5] | 38.3 [43.59-40.7] |  | 2.14 [1.82-2.50] | <0.001 |
| **Alcohol reduction attempts^2^** | |  |  |  |  |  |
|  | May-February | 14.2 [13.1-15.2] | 15.2 [14.1-16.3] |  | - | - |
|  | April | 16.6 [13.0-20.8] | 28.5 [25.0-32.3] |  | 1.94 [1.36-2.75] | <0.001 |
| **Use of evidence-based support^3a^** | |  |  |  |  |  |
|  | May-February | 3.4 [2.1-5.1] | 3.4 [2.2-5.1] |  | - | - |
|  | April | 9.8 [3.7-20.2] | 1.2 [0.1-4.1] |  | 0.18 [0.03-1.06] | 0.058 |
| **Use of remote support^3b^** | |  |  |  |  |  |
|  | May-February | 3.6 [2.2-5.3] | 5.9 [4.2-8.1] |  | - | - |
|  | April | 6.6 [1.8-16.0] | 6.9 [3.6-11.8] |  | 0.89 [0.23-3.41] | 0.868 |
| Note: All data are weighted to match the adult population in England on age, social grade, region, tenure, ethnicity, and working status within sex.  CI, confidence interval. OR_adj_, odds ratio adjusted for age, sex, social grade, region (and, for analyses of use of support, full AUDIT score as an indicator of dependence), trend within year (i.e. May=1 through April=12) and trend across years (i.e. May 2018=1 through April 2020=24).  ^1^ Among all adults (2018/19: May-Feb *n*=17,128, April *n*=1,655; 2019/20: May-Feb *n*=17,126, April *n*=1,649).  ^2^ Among high-risk drinkers (2018/19: May-Feb *n*=4,372, April *n*=373; 2019/20: May-Feb *n*=4,215, April *n*=606).  ^3^ Among high-risk drinkers who made a reduction attempt (2018/19: May-Feb *n*=619, April *n*=61; 2019/20: May-Feb *n*=642, April *n*=173).  ^a^ Prescription medication or face-to-face behavioural support.  ^b^ Telephone support, websites, or apps. | | | | | | |
